# The oxidation‐resistant CaMKII‐MM281/282VV mutation does not prevent arrhythmias in CPVT1

**DOI:** 10.14814/phy2.15030

**Published:** 2021-09-23

**Authors:** Mani Sadredini, Ravinea Manotheepan, Stephan E. Lehnart, Mark E. Anderson, Ivar Sjaastad, Mathis K. Stokke

**Affiliations:** ^1^ Institute for Experimental Medical Research and KG Jebsen Cardiac Research Centre Oslo University Hospital and University of Oslo Oslo Norway; ^2^ Heart Research Center Göttingen Department of Cardiology and Pulmonology University Medical Center Göttingen Georg August University Göttingen Göttingen Germany; ^3^ Cluster of Excellence “Multiscale Bioimaging: from Molecular Machines to Networks of Excitable Cells” (MBExC) University of Göttingen Göttingen Germany; ^4^ DZHK (German Centre for Cardiovascular Research) Göttingen Germany; ^5^ Division of Cardiology Department of Medicine The Johns Hopkins University School of Medicine Baltimore USA; ^6^ Department of Cardiology Oslo University Hospital Rikshospitalet Oslo Norway

**Keywords:** arrhythmias, CaMKII, CPVT, oxidation, RyR2

## Abstract

Catecholaminergic polymorphic ventricular tachycardia type 1 (CPVT1) is an inherited arrhythmogenic disorder caused by missense mutations in the cardiac ryanodine receptors (RyR2), that result in increased β‐adrenoceptor stimulation‐induced diastolic Ca^2+^ leak. We have previously shown that exercise training prevents arrhythmias in CPVT1, potentially by reducing the oxidation of Ca^2+^/calmodulin‐dependent protein kinase type II (CaMKII). Therefore, we tested whether an oxidation‐resistant form of CaMKII protects mice carrying the CPVT1‐causative mutation RyR2‐R2474S (RyR2‐RS) against arrhythmias. Antioxidant treatment (N‐acetyl‐L‐cysteine) reduced the frequency of β‐adrenoceptor stimulation‐induced arrhythmogenic Ca^2+^ waves in isolated cardiomyocytes from RyR2‐RS mice. To test whether the prevention of CaMKII oxidation exerts an antiarrhythmic effect, mice expressing the oxidation‐resistant CaMKII‐MM281/282VV variant (MMVV) were crossed with RyR2‐RS mice to create a double transgenic model (RyR2‐RS/MMVV). Wild‐type mice served as controls. Telemetric ECG surveillance revealed an increased incidence of ventricular tachycardia and an increased arrhythmia score in both RyR2‐RS and RyR2‐RS/MMVV compared to wild‐type mice, both following a β‐adrenoceptor challenge (isoprenaline i.p.), and following treadmill exercise combined with a β‐adrenoceptor challenge. There were no differences in the incidence of arrhythmias between RyR2‐RS and RyR2‐RS/MMVV mice. Furthermore, no differences were observed in β‐adrenoceptor stimulation‐induced Ca^2+^ waves in RyR2‐RS/MMVV compared to RyR2‐RS. In conclusion, antioxidant treatment reduces β‐adrenoceptor stimulation‐induced Ca^2+^ waves in RyR2‐RS cardiomyocytes. However, oxidation‐resistant CaMKII‐MM281/282VV does not protect RyR2‐RS mice from β‐adrenoceptor stimulation‐induced Ca^2+^ waves or arrhythmias. Hence, alternative oxidation‐sensitive targets need to be considered to explain the beneficial effect of antioxidant treatment on Ca^2+^ waves in cardiomyocytes from RyR2‐RS mice.

## Graphical abstract

Inhibition of Ca^2+^/calmodulin‐dependent protein kinase type II (CaMKII) prevents arrhythmias in murine models of catecholaminergic polymorphic ventricular tachycardia (CPVT). CaMKII can be activated by the oxidation of methionines 281/282, and we show that antioxidant treatment may limit arrhythmogenic activity in CPVT. However, genetic inhibition of CaMKII oxidation at methionines 281/282 does not protect CPVT mice from arrhythmias. Other oxidation‐sensitive targets should be explored to explain the effect of antioxidants in CPVT.

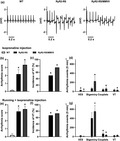

## 1 INTRODUCTION

Catecholaminergic polymorphic ventricular tachycardia (CPVT) is an inherited disorder characterized by β‐adrenoceptor stimulation‐induced ventricular tachycardia (VT) (Baltogiannis et al., 2019). The most common disease‐causing mutations occur in the cardiac ryanodine receptor (RyR2), and cause CPVT type 1. In CPVT, β‐adrenoceptor stimulation‐induced diastolic Ca^2+^ release through the RyR2 triggers spontaneous action potentials that can result in VT. If untreated, CPVT has a mortality rate of 30%–50% by the age of 40 (Wleklinski et al., 2020). Although current therapies, including β‐adrenoceptor antagonists, flecainide, and cardiac sympathetic denervation, highly improve prognosis, treatment of CPVT remains a challenge (Baltogiannis et al., 2019). Therefore, novel therapeutic solutions are being explored (Wleklinski et al., 2020). One promising therapeutic target is Ca^2+^/calmodulin‐dependent protein kinase type II (CaMKII) (Wleklinski et al., 2020). CaMKII is activated downstream of β‐adrenoceptor stimulation by the autophosphorylation of CaMKII‐threonine 286 (T286) (Bezzerides et al., 2019; Mustroph et al., 2017; Xu et al., 2020), but other mechanisms for CaMKII activation have also been demonstrated. This includes O‐glycosylation, S‐nitrosylation, and oxidation (Beckendorf et al., 2018; Erickson et al., 2008, 2013, 2015). Oxidation, in particular, may be relevant for CPVT, since β‐adrenoceptor activation can increase reactive oxygen species (Corbi et al., 2013), and increased reactive oxygen species derived from mitochondria have been found in CPVT (Hamilton et al., 2020). Specifically, oxidation of CaMKII on methionines 281/282 (MM281/282) activates this kinase and can promote arrhythmias in other conditions (Anderson, 2015). CaMKII‐dependent phosphorylation of RyR2 on serine 2814 (S2814) increases diastolic Ca^2+^ release from RyR2, possibly by structural unzipping between N‐terminal and central domains (Uchinoumi et al., 2016). Inhibition of CaMKII can, therefore, protect against diastolic Ca^2+^ release and prevent arrhythmias, as illustrated in murine models of CPVT (Bezzerides et al., 2019; Liu et al., 2011). Furthermore, it has been proposed that CaMKII‐dependent phosphorylation of RyR2 might even be necessary for β‐adrenoceptor stimulation‐induced arrhythmias in CPVT (Park et al., 2019).

Although the inhibition of CaMKII seems like a promising treatment strategy in CPVT, this strategy might have unappreciated effects on health and disease (Beckendorf et al., 2018). This includes hampered cardiac contractile response to exercise training (Burgos et al., 2017) and increased afterload (Reil et al., 2020), as well as adverse effects in other organs (Beckendorf et al., 2018). Thus, increased understanding of how the regulation of CaMKII contributes to the arrhythmogenic phenotype in CPVT might help develop new treatment strategies to prevent arrhythmias, while preserving the physiological effects of CaMKII.

We have previously shown that exercise training can prevent β‐adrenoceptor stimulation‐induced arrhythmias in mice with the CPVT1‐causative mutation RyR2‐R2474S (RyR2‐RS) (Manotheepan et al., 2016). Furthermore, we found exercise training to reduce the phosphorylation of RyR2 on the CaMKII‐specific phosphorylation site S2814. This phosphoregulation was accompanied by attenuated oxidation of CaMKII and diminished oxidative stress, as indicated by a decrease in malondialdehyde levels. Therefore, we hypothesized that the genetic inhibition of CaMKII oxidation in CPVT1 could protect against β‐adrenoceptor stimulation‐induced arrhythmias. To test this, we have crossbred mice with the oxidation‐resistant CaMKII mutation MM281/282VV with RyR2‐RS mice.

## 2 MATERIALS AND METHODS

### 2.1 Ethics statement

The use of animals in this study was approved by the Norwegian Food Safety Authorities (FOTS ID 7798 and 17905), which conforms to the European Union Directive for the protection of experimental animals 2010/63/EU. Animals were housed in cages with a 12 h light/dark cycle and had ad libitum access to food and water.

### 2.2 Animals

Mice with the knock‐in CaMKIIδ‐MM281/282VV mutation (MMVV) with M281 and M282 are replaced with valines to prevent oxidation (Luo et al., 2013) were backcrossed to C57BL/6N for five generations. Importantly, we ensured that the backcrossed mice did not have the nicotinamide nucleotide transhydrogenase (NNT) mutation that is found in C57BL/6J mice (Ronchi et al., 2013). Following backcrossing, these mice were crossed with C57BL/6N mice with the CPVT1‐causative mutation RyR2‐R2474S (RyR2‐RS) (Lehnart et al., 2008) to create mice heterozygous for the RyR2‐RS and homozygous for the MMVV mutation (RyR2‐RS/MMVV). C57BL/6N wild‐type mice (WT) and C57BL/6N MMVV mice served as controls. Age‐ and gender‐matched 2–5 months old female and male mice were used for experiments.

### 2.3 Telemetric ECG surveillance

Buprenorphine 0.1 mg/kg s.c. and bupivacaine 1.8 mg/kg s.c. were administered prior to surgery for analgesia. Anesthesia was induced in an induction chamber with 5% isoflurane and 95% oxygen. Anesthesia was maintained using a nose cone delivering 2% isoflurane and 98% oxygen. A dorsal incision was made and a telemetric ECG transmitter (ETA‐F10, Data Sciences International) was implanted subcutaneously in the lumbar back of the mouse. The ECG electrodes were placed subcutaneously on each lateral side of the thoracic wall. One week was allowed for recovery from the surgery before ECG recordings. A stress test was performed using i.p. administration of 20 mg/kg isoprenaline (in 0.9% saline, pH adjusted to 7.4; isoprenaline hydrochloride, Sigma Aldrich). One week was allowed for recovery from the stress test before a second stress test was conducted consisting of treadmill running followed by isoprenaline administration. Before the second stress test, mice were acclimatized to the treadmill (Columbus Instruments) for 15 min per day for three consecutive days at low speeds (gradually increased to 9 m/min). The stress test was initiated with a speed of 3.6 m/min and increased stepwise by 1.8 m/min every 1.5 min until the mice were unable to continue running (defined as >1 s off the treadmill belt). Isoprenaline was administered promptly after the discontinuation of running. Arrhythmias occurring during the first five minutes following isoprenaline administration were analyzed using Ponemah software (Data Sciences International). An arrhythmia score based on the most severe arrhythmia was applied: no arrhythmias = 0; isolated ventricular extrasystoles (VES) = 1; VES in bigeminy = 2; couplets = 3; VT = 4. VT was defined as three or more consecutive VES. Investigators were blinded to the animal genotype during both experiments and analyses.

### 2.4 Cell isolation

Before anesthesia induction, ~50 IU heparin/20 g i.p. was administered. Anesthesia induction and maintenance were conducted in the same manner as described under Telemetric ECG surveillance. Following thoracotomy, the heart was excised and put in ice‐cold isolation buffer (in mM: 130 NaCl, 25 HEPES, 22 glucose, 5.4 KCl, 0.5 MgCl_2_, 0.4 NaH_2_PO_4_; pH adjusted to 7.4 with NaOH). The heart was then mounted on a modified Langendorff setup and perfused with isolation buffer at 37℃. The perfusion solution was gradually switched to an isolation buffer containing 0.012 mM Ca^2+^ and ~500 U/mL of collagenase type II (Worthington). Following digestion for 6–10 min, the left ventricle was isolated, transferred into isolation buffer with 0.02 mM Ca^2+^ and 0.1% bovine serum albumin, cut into pieces, and triturated with ~100 U/mL of deoxyribonuclease I (Worthington). The solution was filtered through a Nitex mesh with a pore size of 255 µm and the Ca^2+^ concentration increased stepwise every ~4 min to 0.05, 0.1, 0.2, and 0.5 mM. Cardiomyocytes were kept at room temperature and used within 6 h.

### 2.5 Whole‐cell Ca^2+^ imaging

Cardiomyocytes were incubated with 2 µM of the Ca^2+^ indicator Fluo‐4 AM ester (Invitrogen) for 15 min including 5 min on laminin‐coated coverslips mounted in a perfusion chamber. Cardiomyocytes were superfused with a modified HEPES‐buffered Tyrode's solution (in mM: 140 NaCl, 5.5 glucose, 5.4 KCl, 5 HEPES, 1 CaCl_2_, 0.5 MgCl_2_, 0.4 NaH_2_PO_4_; pH adjusted to 7.4 with NaOH) at 37 ± 0.5℃. A Zeiss Axio Observer microscope with a PTI D‐104 microscope photometer and PTI Felix software were applied to record whole‐cell Ca^2+^ fluorescence intensity. Rod‐shaped cardiomyocytes without membrane blebs were paced using platinum electrodes at ~20% above individual threshold for contraction at either 1 or 4 Hz for 20 s. Ca^2+^ waves were studied in a 10 s post‐pacing rest period. If no Ca^2+^ waves occurred in the rest period, Ca^2+^ wave latency was set to 10 s. Sarcoplasmic reticulum (SR) Ca^2+^ content was estimated from the Ca^2+^ release evoked by the application of 10 mM caffeine (Sigma Aldrich) following 20 s of 1 Hz pacing. The effects of β‐adrenoceptor stimulation were assessed following the application of 100 nM isoprenaline (ISO; isoprenaline sulfate, NAF, Norway) for a minimum of 100 s. Ca^2+^ reuptake rate was measured as the rate constant of the Ca^2+^ transient decay (1/tau) using ClampFit software (v 10.4.1.0, Axon Instruments, Inc). SR Ca^2+^ ATPase (SERCA)‐dependent Ca^2+^ reuptake was calculated by subtracting the rate constant of the caffeine transient from the rate constant of the Ca^2+^ transient. To study the effect of antioxidant treatment on Ca^2+^ waves in RyR2‐RS, cardiomyocytes were preincubated with 5 mM N‐acetyl‐L‐cysteine (NAC) for 20 min prior to superfusion with the HEPES‐buffered Tyrode's solution with 5 mM NAC added. Investigators were blinded to the treatment with NAC (vs. vehicle), or to the animal genotype, during both experiments and analyses.

### 2.6 Immunoblotting

Anesthesia was induced and maintained as described under Telemetric ECG surveillance. A thoracotomy was performed and the heart was excised and put in ice‐cold saline. To study the effects of β‐adrenoceptor stimulation on phosphoprotein status 20 mg/kg isoprenaline (in 0.9% saline, pH adjusted to 7.4; isoprenaline hydrochloride, Sigma Aldrich) was administered i.p. 3 min before excision of the heart in a cohort of mice. The left ventricle was isolated, snap‐frozen in liquid nitrogen, and stored at −80℃. The frozen tissue was homogenized in lysis buffer (1% Triton, 0.1% Tween, 99% 1x PBS) using a Polytron 1200 homogenizer. Phosphatase and protease inhibitor cocktails (Roche Diagnostics) were added. Micro BCA protein assay kit (Thermo Fisher Scientific) was used to quantify protein concentrations. Sample buffer (50% sucrose, 7.5% SDS, 62.5 mM Tris–HCl, 2 mM EDTA, 3.1% DTT, and 0.01% bromophenol blue) was added to the protein extract followed by heating at 94℃ for 5 min. The samples were size fractioned on Criterion 4–15% Tris–HCl gels (15% for phospholamban) and transferred to 0.2 µm PVDF‐membranes (Bio‐Rad Laboratories). Blots were blocked in 3% BSA or 5% non‐fat milk in Tris‐buffered saline with 1% Tween and incubated with primary antibodies overnight and secondary antibodies for one hour at room temperature. The following antibodies were used: anti‐CaMKII phospho T286 (ab32678, Abcam plc., Cambridge, UK), anti‐RyR2 phospho S2808 (A010‐30), anti‐RyR2 phospho S2814 (A010‐31), anti‐phospholamban phospho S16 (A010‐12), anti‐phospholamban phospho T17 (A010‐13) (Badrilla Ltd, Leeds, UK), anti‐phospholamban (MA3‐922), anti‐CaMKIIδ (PA5‐22168), anti‐RyR2 (MA3‐916) (Thermo Fisher Scientific Inc.), anti‐GAPDH (Sc‐20357, Santa Cruz Biotechnology), anti‐vinculin (V9131, Sigma‐Aldrich). Anti‐goat, anti‐rabbit or anti‐mouse IgG horseradish peroxidase‐conjugated antibody (R&D Systems; GE Healthcare) was used as the secondary antibody. Amersham ECL Prime (GE Healthcare) was used to develop blots and signals were detected by LAS‐4000 (Fujifilm Life Science).

### 2.7 Statistics

Results are reported as the mean ± standard error of the mean. A Nested ANOVA using the lme function in R software for statistical programming was used for comparisons of all data from isolated cardiomyocytes (Manotheepan et al., 2016). Two‐way ANOVA was used to combine both pacing frequencies to test the effect of NAC on Ca^2+^ wave frequency. Fisher's exact test was used to study the proportion of animals developing VT. Kruskal–Wallis test was used to compare arrhythmia scores and arrhythmia events. Unpaired t‐test was used for comparison of phosphoproteins.

## 3 RESULTS

### 3.1 Antioxidant treatment reduces arrhythmogenic Ca^2+^ waves in RyR2‐RS

**FIGURE 1:**
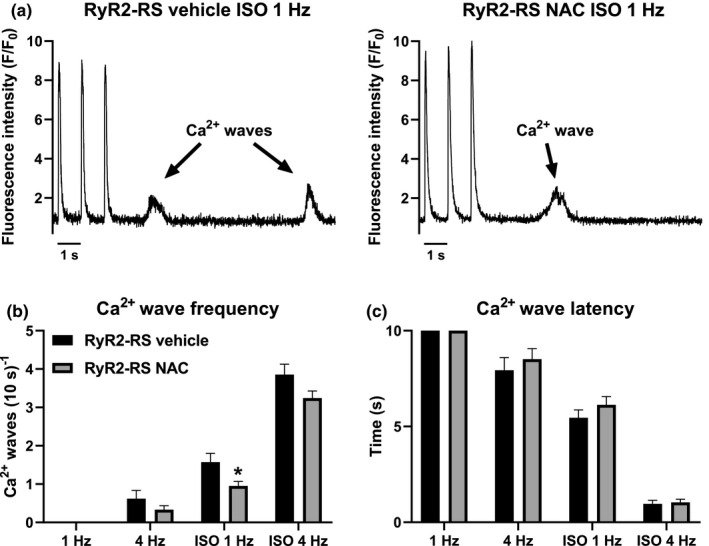
Antioxidant treatment reduces Ca^2+^ waves: (a) Illustrative tracings from a vehicle‐treated RyR2‐RS cardiomyocyte (left) paced at 1 Hz during β‐adrenoceptor stimulation with 100 nM ISO showing Ca^2+^ waves (indicated by arrows) in the post‐pacing rest period. Illustrative tracing from a NAC‐treated RyR2‐RS cardiomyocyte (right) during the same experimental conditions. (b) The frequency of Ca^2+^ waves was measured in a 10 s post‐pacing rest period in cardiomyocytes paced at 1 and 4 Hz under baseline conditions and during β‐adrenoceptor stimulation (ISO). (c) The time from the beginning of the last paced Ca^2+^ transient to the beginning of the first Ca^2+^ wave was analyzed, that is, Ca^2+^ wave latency. Nested ANOVA; *p = 0.024 compared to the vehicle; 11 hearts, 20–23 cells per group at baseline and 62–65 cells per group during ISO stimulation

We tested whether antioxidant treatment could prevent arrhythmogenic Ca^2+^ waves in CPVT1. RyR2‐RS cardiomyocytes pretreated with the antioxidant NAC were paced at 1 and 4 Hz with a post‐pacing rest period to study Ca^2+^ waves (Figure 1a). Under baseline conditions, that is, without β‐adrenoceptor stimulation, no Ca^2+^ waves were detected following 1 Hz pacing in either NAC‐treated or vehicle‐treated cardiomyocytes (Figure 1b). Furthermore, only a few Ca^2+^ waves occurred following 4 Hz stimulation, and no difference in the frequency or latency of Ca^2+^ waves were observed in NAC‐treated compared to vehicle‐treated RyR2‐RS cardiomyocytes (Figure 1b–c). Arrhythmias in CPVT are induced by β‐adrenoceptor stimulation; therefore, we next studied the effect of NAC on Ca^2+^ waves during stimulation with the β‐adrenoceptor agonist ISO. In these conditions, NAC reduced the frequency of Ca^2+^ waves following 1 Hz pacing in RyR2‐RS cardiomyocytes compared to the vehicle but had no effect on Ca^2+^ wave latency (Figure 1b–c). NAC did not affect Ca^2+^ wave latency or Ca^2+^ wave frequency following 4 Hz stimulation during β‐adrenoceptor stimulation (Figure 1b–c). However, a two‐way ANOVA combining both pacing frequencies during β‐adrenoceptor stimulation indicated reduced Ca^2+^ wave frequency in NAC‐treated cardiomyocytes (2.1 ± 0.2 vs. 2.7 ± 0.2, *p *= 0.004). These data indicate that the inhibition of oxidation by antioxidant treatment may protect against arrhythmogenic Ca^2+^ release in CPVT1.

### 3.2 CPVT1 mice with oxidation‐resistant CaMKII are not protected from arrhythmias

**FIGURE 2:**
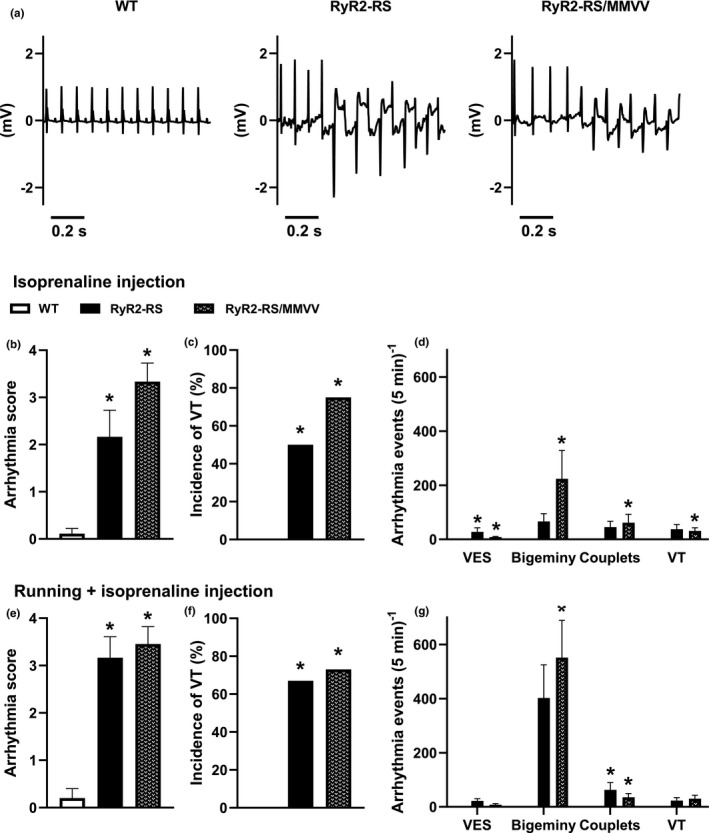
Genetic inhibition of CaMKII MM281/282 oxidation does not affect arrhythmias in CPVT1: (a) Illustrative ECG tracings from a WT, a RyR2‐RS, and a RyR2‐RS/MMVV mouse following i.p. administration of 20 mg/kg ISO. The WT tracing shows sinus rhythm, while the tracings from RyR2‐RS and RyR2‐RS/MMVV show a few sinus rhythms preceding bidirectional VT. (b) Arrhythmias were studied for 5 min following isoprenaline (ISO) administration. An arrhythmia score was used based on the severity of arrhythmias. (c) The percentage of animals that developed the most severe arrhythmia, that is, VT. (d) The numbers of the subtypes of arrhythmias counted in 5 min following ISO administration. The experiment was repeated with treadmill running preceding the ISO administration with (e) arrhythmia score, (f) percentage of animals with VT and (g) subtypes of arrhythmias shown. Kruskal–Wallis test for arrhythmia score and arrhythmia events; Fisher's exact test for incidence of VT; *p < 0.05 compared to WT; 9 WT, 12 RyR2‐RS, and 12 RyR2‐RS/MMVV animals for isoprenaline only; 5 WT, 12 RyR2‐RS, and 11 RyR2‐RS/MMVV animals for running + ISO

Next, we specifically tested whether preventing CaMKII oxidation on the MM281/282 oxidation site could protect CPVT1 mice from β‐adrenoceptor stimulation‐induced arrhythmias. For this purpose, arrhythmias were studied in RyR2‐RS/MMVV, RyR2‐RS, and WT mice following i.p. administration of 20 mg/kg isoprenaline (Figure 2a). As expected, RyR2‐RS mice exhibited an increased incidence of ventricular arrhythmias indicated by increased arrhythmia score compared to WT (Figure 2b). Furthermore, 50% of the RyR2‐RS mice developed VT (6/12), that is, the most severe arrhythmia observed, while no such arrhythmias were observed in WT mice (0/9; Figure 2c). Surprisingly, RyR2‐RS/MMVV mice showed a similar increase in arrhythmia score and incidence of VT compared to WT (9/12, Figure 2b–c). Consistent with this, we did not observe any difference in arrhythmia score or incidence of VT between RyR2‐RS/MMVV and RyR2‐RS mice (Figure 2b–c). Moreover, analysis of the arrhythmia subtypes did not reveal any difference between RyR2‐RS/MMVV and RyR2‐RS (Figure 2d). To ensure adequate β‐adrenoceptor stimulation and consistency with our previous work (Manotheepan et al., 2016), we repeated this experiment with treadmill running in addition to the administration of isoprenaline. Similar to the previous test, both RyR2‐RS and RyR2‐RS/MMVV showed increased arrhythmia score and increased incidence of VT compared to WT (Figure 2e–f). Furthermore, we did not find any difference in arrhythmia score, incidence of VT or subtypes of arrhythmia between RyR2‐RS/MMVV and RyR2‐RS (Figure 2e–g). Thus, our data show that preventing the oxidation of CaMKII at the MM281/282 oxidation site does not influence the susceptibility to β‐adrenoceptor stimulation‐induced arrhythmias in CPVT1.

### 3.3 Oxidation‐resistant CaMKII does not prevent Ca^2+^ waves in CPVT1

**FIGURE 3:**
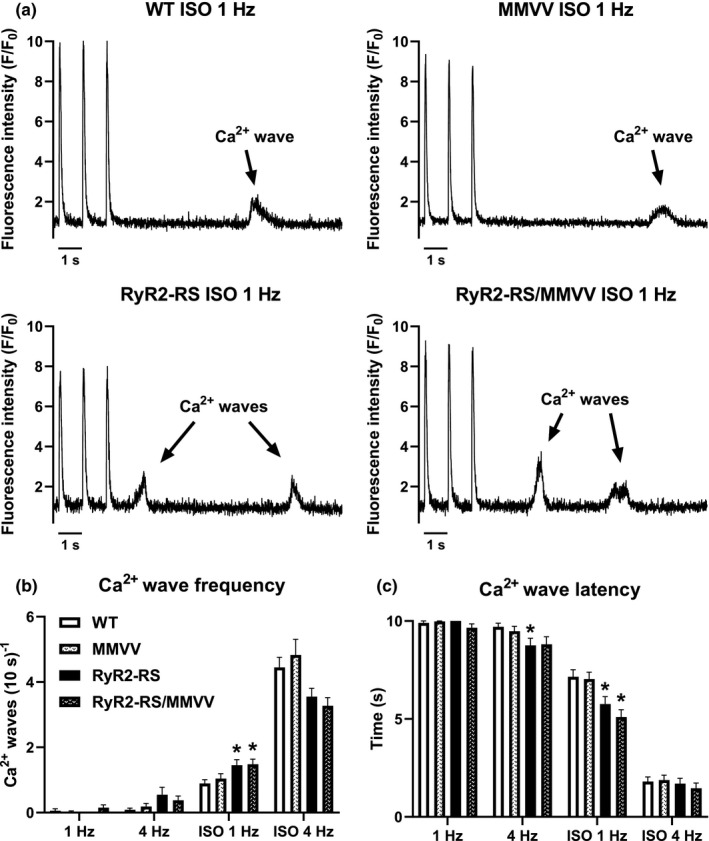
Oxidation‐resistant CaMKII does not affect Ca^2+^ waves in CPVT1: (a) Illustrative tracings from WT, MMVV, RyR2‐RS, and RyR2‐RS/MMVV cardiomyocytes with paced Ca^2+^ transients at 1 Hz during ISO stimulation (100 nM ISO) and post‐pacing rest period with Ca^2+^ waves indicated by arrows. (b) Frequency of Ca^2+^ waves at baseline and during ISO stimulation. (c) Latency of Ca^2+^ waves at baseline and during ISO stimulation. Nested ANOVA; all genotypes were compared to WT, RyR2‐RS/MMVV was also compared to RyR2‐RS; *p < 0.05 compared to WT; 15–16 animals in each group; 37–53 cells per group at baseline; 84–95 cells per group during ISO stimulation

Although the in vivo data do not show any effect of oxidation‐resistant CaMKII on arrhythmias, this could be influenced by the effects of CaMKIIδ in other cells than cardiomyocytes, such as neurons (Ye et al., 2019). In vitro phenotyping may help assess the contribution of cardiomyocytes to the in vivo phenotype. Therefore, we studied the effect of oxidation‐resistant CaMKII on arrhythmogenic Ca^2+^ waves in isolated cardiomyocytes (Figure 3a). Here, in addition to WT, RyR2‐RS, and RyR2‐RS/MMVV, we included cardiomyocytes from MMVV mice to uncover if this mutation has any proarrhythmic effects independent of the CPVT1 mutation. Under baseline conditions, few Ca^2+^ waves were detected following 1 and 4 Hz pacing, with no difference in Ca^2+^ wave frequency between the groups (Figure 3b). However, Ca^2+^ waves did occur with shorter latency in RyR2‐RS cardiomyocytes following 4 Hz pacing compared to WT (Figure 3c). During β‐adrenoceptor stimulation, both RyR2‐RS and RyR2‐RS/MMVV showed increased Ca^2+^ wave frequency and shortened Ca^2+^ wave latency following 1 Hz pacing compared to WT (Figure 3b–c). Importantly, no difference in Ca^2+^ wave frequency or latency was observed between RyR2‐RS and RyR2‐RS/MMVV following 1 and 4 Hz pacing during β‐adrenoceptor stimulation (Figure 3b–c). Noteworthy, MMVV did not differ from WT in either frequency or latency of Ca^2+^ waves (Figure 3b–c). These data imply that the genetic inhibition of CaMKII oxidation does not prevent arrhythmogenic Ca^2+^ waves in CPVT1.

### 3.4 Oxidation of CaMKII has limited effects on Ca^2+^ handling in CPVT1

**FIGURE 4:**
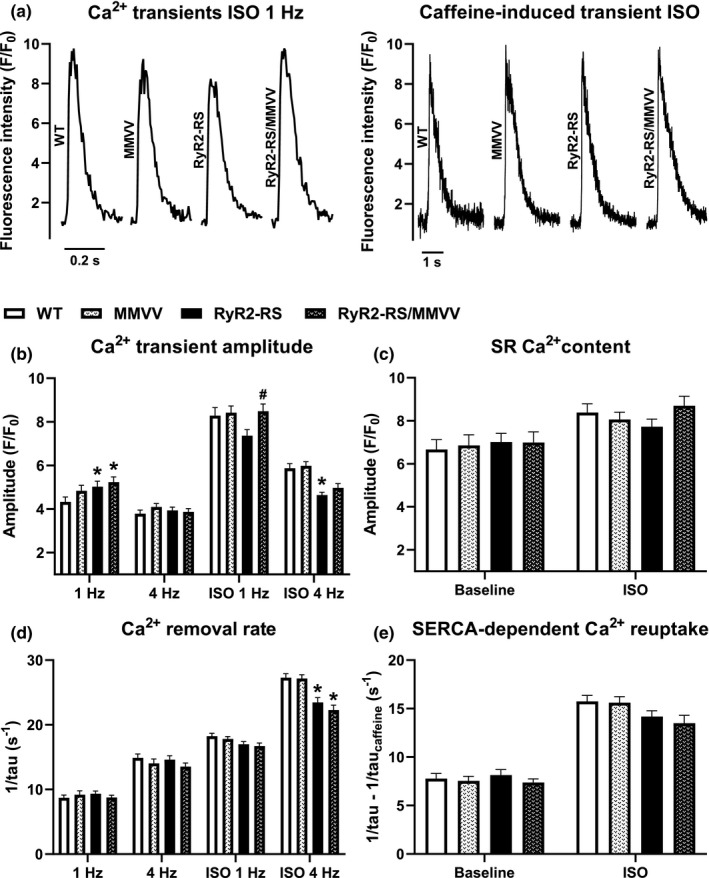
The effect of oxidation‐resistant CaMKII on Ca^2+^ handling in CPVT1: (a) Illustrative tracings from 1 Hz‐paced Ca^2+^ transients (left) and caffeine‐induced transients (right), both during β‐adrenoceptor stimulation with 100 nM ISO. (b) Amplitude of the paced Ca^2+^ transients at 1 and 4 Hz, under baseline conditions and during β‐adrenoceptor stimulation (ISO). (c) SR Ca^2+^ content following 1 Hz pacing measured as the amplitude of the caffeine‐induced transient (10 mM caffeine), at baseline and during ISO stimulation. (d) Exponential fitting to the decay of the Ca^2+^ transient was used to assess the Ca^2+^ removal rate. (e) SERCA‐dependent Ca^2+^ reuptake was calculated by subtracting the decay rate constant of the caffeine‐induced transient (following 1 Hz pacing) from the decay rate constant of the paced Ca^2+^ transient at 1 Hz from the same cardiomyocyte. Nested ANOVA; all genotypes were compared to WT, RyR2‐RS/MMVV was also compared to RyR2‐RS; *p < 0.05 compared to WT; ^#^ *p *< 0.05 compared to RyR2‐RS; 15–16 animals in each group; 38–53 cells for Ca^2+^ transients and 21–28 cells for caffeine transients at baseline, 79–97 cells for Ca^2+^ transients and 27–71 for caffeine transients during ISO stimulation

Although oxidation‐resistant CaMKII did not affect arrhythmogenic Ca^2+^ release in RyR2‐RS, it could affect other aspects of Ca^2+^ handling. Thus, we studied paced Ca^2+^ transients and caffeine‐induced Ca^2+^ transients (representative tracings during β‐adrenoceptor stimulation shown in Figure 4a). At baseline during 1 Hz pacing, both RyR2‐RS and RyR2‐RS/MMVV cardiomyocytes showed increased Ca^2+^ transient amplitude compared to WT (Figure 4b). However, we observed no differences between RyR2‐RS/MMVV and RyR2‐RS or between MMVV and WT (Figure 4b). Furthermore, the genotypes did not affect the Ca^2+^ transient amplitude during 4 Hz pacing. During β‐adrenoceptor stimulation the Ca^2+^ transient amplitudes at 1 Hz pacing were not altered in MMVV, RyR2‐RS or RyR2‐RS/MMVV compared to WT (Figure 4b). Interestingly, RyR2‐RS/MMVV showed increased Ca^2+^ transient amplitude compared to RyR2‐RS at 1 Hz pacing during β‐adrenoceptor stimulation (Figure 4b). Moreover, at 4 Hz pacing during β‐adrenoceptor stimulation, RyR2‐RS showed reduced Ca^2+^ transient amplitude compared to WT, while no such difference was observed in MMVV and RyR2‐RS/MMVV compared to WT (Figure 4b). In contrast to 1 Hz pacing, at 4 Hz pacing during β‐adrenoceptor stimulation, RyR2‐RS/MMVV Ca^2+^ transient amplitudes did not differ from RyR2‐RS (Figure 4b). The amplitude of the Ca^2+^ transient depends on the SR Ca^2+^ content which can be altered by both CaMKII and RyR2 function (Mustroph et al., 2017). Surprisingly, we did not find any changes in SR Ca^2+^ content (measured as the amplitude of the caffeine‐induced Ca^2+^ transient, Figure 4a right panel) between the groups, both under baseline conditions and during β‐adrenoceptor stimulation (Figure 4c). Nevertheless, CaMKII‐dependent phosphorylation of phospholamban (PLB) can increase SERCA activity and thereby cytosolic Ca^2+^ removal (Mustroph et al., 2017). We found that cardiomyocytes with the CPVT1 mutation had lower Ca^2+^ removal rate at 4 Hz pacing during β‐adrenoceptor stimulation (i.e., reduced Ca^2+^ removal rate in both RyR2‐RS and RyR2‐RS/MMVV compared to WT, Figure 4d). However, the MMVV mutation did not influence the Ca^2+^ removal rate (i.e., no significant difference between MMVV and WT, or between RyR2‐RS/MMVV and RyR2‐RS, Figure 4d). Furthermore, we did not find any differences in SERCA‐dependent Ca^2+^ reuptake between the groups, either at baseline or during β‐adrenoceptor stimulation (Figure 4e). Thus, genetic inhibition of CaMKII oxidation seems to have only minor effects on Ca^2+^ handling in CPVT1.

### 3.5 Oxidation of CaMKII does not contribute to RyR2 phosphorylation in CPVT1

**FIGURE 5:**
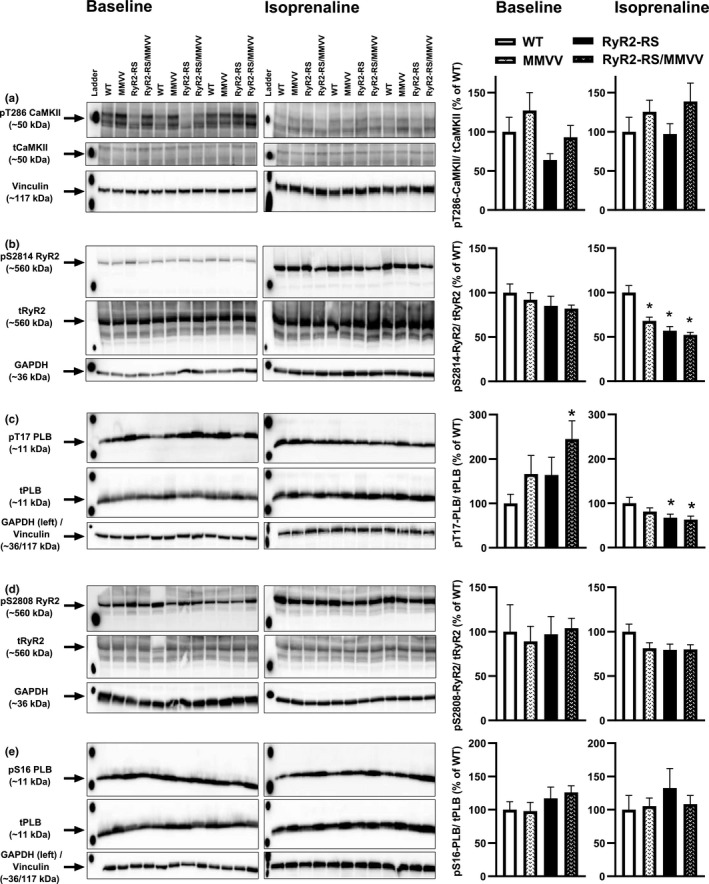
The effect of oxidation‐resistant CaMKII on Ca^2+^ handling phosphoproteins: Immunoblots were performed on left ventricular homogenates from untreated mice (baseline) and mice three min after the administration of 20 mg/kg isoprenaline i.p. (isoprenaline). (a) Illustrative immunoblots of phosphorylated CaMKII on T286, total CaMKII and vinculin as loading control with averaged data for T286 phosphorylated CaMKII normalized to total CaMKII. (b) Illustrative immunoblots of phosphorylated RyR2 on the CaMKII phosphorylation site S2814, total RyR2, and GAPDH as loading control with averaged data for S2814‐RyR2 normalized to total RyR2. (c) Illustrative immunoblots of phosphorylated PLB on T17, total PLB, and GAPDH or vinculin as loading controls with averaged data for T17‐PLB normalized to total PLB. (d) Illustrative immunoblots of RyR2 phosphorylation on the protein kinase A phosphorylation site S2808, total RyR2, and GAPDH as loading control with averaged data for S2808 normalized to total RyR2. (e) Illustrative immunoblots of phosphorylated PLB on S16, total PLB, and GAPDH or vinculin as loading control with averaged data for S16‐PLB normalized to total PLB are shown. T‐test; all genotypes were compared to WT, RyR2‐RS/MMVV was also compared to RyR2‐RS; *p < 0.05; 5 WT, 8 MMVV, 8 RyR2‐RS, and 8 RyR2‐RS/MMVV mice

Last, we studied CaMKII, RyR2, and PLB phosphorylation status in left ventricles from both untreated mice (i.e., baseline conditions) and in mice exposed to i.p. administration of isoprenaline three min before harvest to examine the effects of β‐adrenoceptor stimulation (Figure 5). RyR2‐RS/MMVV was compared to RyR2‐RS, while MMVV, RyR2‐RS, and RyR2‐RS/MMVV were all compared to WT in both conditions. Of note, the experimental setup did not allow direct comparison between untreated and isoprenaline‐treated hearts. As expected, CaMKII autophosphorylation on T286 was not significantly different in the genotypes neither at baseline nor following β‐adrenoceptor stimulation (Figure 5a). Oxidation‐dependent CaMKII activation can lead to the phosphorylation of RyR2 on S2814 (Anderson, 2015). Under baseline conditions, we did not detect any significant difference in phosphorylation at this site between the genotypes (Figure 5b). Interestingly, at baseline, CaMKII‐dependent phosphorylation of PLB at T17 was higher in RyR2‐RS/MMVV than in WT, but no differences were found in the other comparisons (Figure 5c). Following β‐adrenoceptor stimulation, RyR2‐S2814 phosphorylation was lower in MMVV, RyR2‐RS, and RyR2‐RS/MMVV than in WT (Figure 5b). This coincided with a lower CaMKII‐dependent PLB‐T17 phosphorylation in RyR2‐RS and RyR2‐RS/MMVV than in WT (Figure 5c). Importantly, no significant differences were observed between RyR2‐RS/MMVV and RyR2‐RS in either RyR2‐S2814 or PLB‐T17 phosphorylation. Protein kinase A‐dependent phosphorylation of RyR2‐S2808 and PLB‐S16 were not significantly different between the genotypes neither at baseline nor following β‐adrenoceptor stimulation (Figure 5d and e).

## 4 DISCUSSION

CaMKII is a promising target for arrhythmia prevention in CPVT. Increased understanding of how CaMKII contributes to the arrhythmogenic phenotype in CPVT can help bring this strategy closer to clinical use. Here, we find that the oxidation‐resistant CaMKII MM281/282VV mutation does not prevent arrhythmias or arrhythmogenic Ca^2+^ waves in CPVT1.

### 4.1 Oxidation of CaMKII as a target in CPVT1

Growing evidence supports the importance of CaMKII MM281/282 oxidation in the development of arrhythmias (Himelman et al., 2020; Ho et al., 2014; Purohit et al., 2013). CaMKII oxidation could also play a role in the pathophysiology of CPVT. For instance, increased mitochondria‐derived reactive oxygen species have been found in CPVT (Hamilton et al., 2020) and could potentiate arrhythmogenic Ca^2+^ release through the oxidation of CaMKII. We have previously shown that exercise training reduces CaMKII oxidation, CaMKII‐dependent RyR2‐S2814 phosphorylation, arrhythmogenic Ca^2+^ release, and arrhythmias in the RyR2‐RS CPVT1 mouse model (Manotheepan et al., 2016). In addition, we ruled out CaMKII autophosphorylation on T286 as the cause of reduced RyR2‐S2814 phosphorylation following exercise training (Manotheepan et al., 2016). Here, we find that the genetic inhibition of CaMKII MM281/282 oxidation results in less RyR2‐S2814 phosphorylation following β‐adrenoceptor stimulation than seen in WT. However, CaMKII autophosphorylation was not affected by the MM281/282VV construct, neither at baseline nor following β‐adrenoceptor stimulation. Thus, the lower CaMKII‐dependent RyR2‐S2814 phosphorylation in the MMVV mice following β‐adrenoceptor stimulation is likely a result of lower CaMKII activity due to reduced MM281/282 oxidation. The observation that β‐adrenoceptor stimulation is necessary to reveal this effect of the MM281/282VV construct on RyR2‐S2814 phosphorylation may suggest that the oxidative activation of CaMKII accounts for some of the kinase activity provoked by β‐adrenoceptor stimulation. Surprisingly, following β‐adrenoceptor stimulation, the CPVT1 mice also show lower RyR2‐S2814 phosphorylation than WT. Our data show that the genetic inhibition of CaMKII oxidation does not add further limitations in RyR2‐S2814 phosphorylation in the CPVT1 mice. This explains why the MM281/282VV mutation fails to reduce arrhythmias and Ca^2+^ waves in the CPVT1 mice. We can only speculate why the CPVT1 mice show lower CaMKII‐dependent phosphorylation of RyR2‐S2814 compared to WT following β‐adrenoceptor stimulation. This may be a compensatory mechanism to prevent excessive SR Ca^2+^ leak, although not enough to prevent arrhythmias. Such compensatory mechanisms could involve increased phosphatase activity or alternative CaMKII regulatory mechanisms. However, whether exercise training reduced RyR2‐S2814 phosphorylation by reducing the oxidation of CaMKII in our previous work remains unanswered. It is possible that the small physiological reduction in CaMKII oxidation following exercise exerts a different effect on CaMKII activity and thereby RyR2 phosphorylation compared to the genetic replacement of CaMKII MM281/282 with valines to prevent CaMKII oxidation. It is also possible that mechanisms independent of CaMKII oxidation might have contributed to reduced RyR2‐S2814 phosphorylation in RyR2‐RS following exercise training (Manotheepan et al., 2016). One such mechanism might depend on protein phosphatase 2A, shown to be upregulated in exercise (Qin et al., 2019), which can dephosphorylate RyR2‐S2814 (Terentyev & Hamilton, 2016), and might have contributed to the decreased phosphorylation found in RyR2‐RS following exercise.

Although our data suggest that the oxidation of CaMKII MM281/282 is not involved in the arrhythmogenicity of the current CPVT1 model, we cannot extrapolate to other CPVT1 models. It is possible that there is an insufficient degree of reactive oxygen species under our experimental conditions for the oxidant activation of CaMKII to occur. Importantly, we have used C57BL/6N mice which lack the loss‐of‐function mutation in the antioxidative enzyme NNT that is found in C57BL/6J mice (Ronchi et al., 2013). The intact NNT in C57BL/6N plays an important role in the removal of reactive oxygen species (Ronchi et al., 2013) and may have contributed to insufficient levels of oxidative stress for considerable oxidant activation of CaMKII to take place under our experimental conditions. Noteworthy, the MMVV mice have normal activity of CaMKII in response to T286 autophosphorylation (Erickson et al., 2008). Thus, it is possible that the activation of CaMKII by autophosphorylation at T286 during β‐adrenoceptor stimulation precludes additional oxidation‐dependent CaMKII activity in the RyR2‐RS CPVT1 mice. If this is the case, the prevention of CaMKII MM281/282 oxidation will not be protective in CPVT, as arrhythmias in CPVT occur in situations with increased β‐adrenoceptor activation (Wleklinski et al., 2020).

We find the antioxidant NAC to have a preventive effect on the occurrence of arrhythmogenic Ca^2+^ waves in RyR2‐RS cardiomyocytes. Our data suggest that this effect is not caused by reduced CaMKII MM281/282 oxidation. CaMKII can also be oxidized at M308, however, based on current knowledge, oxidation of this residue would be expected to protect against arrhythmias in CPVT1 (Konstantinidis et al., 2020). Therefore, other redox‐sensitive Ca^2+^ handling proteins than CaMKII are likely involved in the effect of NAC on Ca^2+^ waves (Tse et al., 2016). One obvious candidate is RyR2, as the oxidation of RyR2 itself can contribute to arrhythmogenic Ca^2+^ release (Bovo et al., 2018; Huang et al., 2020; Nikolaienko et al., 2020; Prosser et al., 2011). Indeed, a recent report demonstrates that the oxidation of RyR2 can contribute to the development of Ca^2+^ waves in CPVT, which can be prevented by antioxidant treatment (Hamilton et al., 2020).

### 4.2 Experimental limitations of Ca^2+^ waves

One surprising observation in our study is that we only find increased Ca^2+^ wave frequency in RyR2‐RS compared to WT cardiomyocytes following 1 Hz pacing, and not 4 Hz. Possibly, this could be due to reduced SR Ca^2+^ content following 4 Hz pacing in RyR2‐RS compared to WT. Although, we did not measure SR Ca^2+^ content following 4 Hz pacing, a more pronounced rate‐dependent reduction in SR Ca^2+^ content has been observed in CPVT1 compared to WT (Fernández‐Velasco et al., 2009). Furthermore, both RyR2‐RS and RyR2‐RS/MMVV showed lower Ca^2+^ removal rate compared to WT at 4 Hz pacing during β‐adrenoceptor stimulation. Our best explanation for this is an increase in spontaneous Ca^2+^ release occurring during the SR Ca^2+^ reuptake in RyR2‐RS, thereby slowing Ca^2+^ removal (Guo et al., 2012). We can only speculate whether such early spontaneous release is increased in RyR2‐RS and reduces the likelihood of spontaneous Ca^2+^ waves later in the post‐pacing rest period. Noteworthy, we limited the examination of Ca^2+^ waves to left ventricular cardiomyocytes following pacing which is a generally accepted method (Danielsen et al., 2018; Hwang et al., 2019; Liu et al., 2011; Manotheepan et al., 2016; Sedej et al., 2010; Tan et al., 2016). The small differences in Ca^2+^ wave frequency between CPVT1 and WT cardiomyocytes observed here do not seem to reflect the large effects of the CPVT1 mutation on arrhythmias in vivo (e.g., 50%–75% VT incidence in RyR2‐RS groups compared to 0% in WT). However, the small differences in Ca^2+^ wave frequency are comparable to previous reports (Bonilla et al., 2019; Savio‐Galimberti & Knollmann, 2015). Importantly, in addition to Ca^2+^ waves, Ca^2+^ sparks and “invisible” leak also contribute to the total Ca^2+^ leak (Hoang‐Trong et al., 2015). In particular, we have recently found that Ca^2+^ leak mediated by Ca^2+^ sparks is substantially higher in RyR2‐RS than WT (Sadredini et al., 2021). Furthermore, examination of Ca^2+^ waves during pacing may better reflect the physiological situation (Fernández‐Velasco et al., 2009). Nonetheless, we did not study Purkinje cells, found by some studies to be important for arrhythmias in CPVT (Baher et al., 2011; Cerrone et al., 2007; Herron et al., 2010; Kang et al., 2010).

### 4.3 Altered depolarization‐induced Ca^2+^ release in RyR2‐RS and the effect of resistance to CaMKII oxidation

Under baseline conditions during 1 Hz pacing, we find increased Ca^2+^ transient amplitude in both RyR2‐RS and RyR2‐RS/MMVV compared to WT despite similar SR Ca^2+^ content. This is presumably due to increased fractional Ca^2+^ release caused by increased opening probability in RyR2‐RS (Lascano et al., 2017). In contrast during β‐adrenoceptor stimulation, Ca^2+^ transient amplitude is unaltered in both RyR2‐RS and RyR2‐RS/MMVV at 1 Hz pacing compared to WT and even reduced at 4 Hz pacing in RyR2‐RS compared to WT. The latter is consistent with previous works and often ascribed to reduced SR Ca^2+^ content in CPVT (Fernández‐Velasco et al., 2009; Ferrantini et al., 2016; Kashimura et al., 2010). We do not find a significant reduction in SR Ca^2+^ content following 1 Hz pacing. However, the above‐mentioned rate‐dependent SR Ca^2+^ depletion in CPVT1 (Fernández‐Velasco et al., 2009) may be the cause of reduced Ca^2+^ transient amplitude in RyR2‐RS at 4 Hz pacing. Surprisingly, the Ca^2+^ transient amplitude was increased in RyR2‐RS/MMVV compared to RyR2‐RS at 1 Hz pacing during β‐adrenoceptor stimulation. We did not detect any significant difference in SR Ca^2+^ content between RyR2‐RS/MMVV and RyR2‐RS. We can only speculate whether minor effects of the MM281/282VV mutation on CaMKII targets, such as the L‐type Ca^2+^ channel, have contributed to the increased Ca^2+^ transient amplitude.

## 5 CONCLUSIONS

Our data demonstrate that genetically engineered resistance to CaMKII MM281/282 oxidation does not protect against arrhythmogenic Ca^2+^ release or arrhythmias in CPVT1. Concurrently, we find antioxidant treatment to reduce arrhythmogenic Ca^2+^ release in CPVT1. Future studies are needed to further examine the role of other targets for oxidation to advance new therapeutic strategies for CPVT.

## CONFLICTS OF INTEREST

None declared.

## AUTHOR CONTRIBUTIONS

All authors conceived and designed research; M.S. and R.M. performed experiments; M.S. analyzed data; all authors interpreted results of experiments; M.S. and M.K.S. drafted the manuscript; all authors edited and revised the manuscript; all authors approved the final version of the manuscript.
